# Supplementary material for: Association of Elevated Serum Uric Acid with Nerve Conduction Function and Peripheral Neuropathy Stratified by Gender and Age in Type 2 Diabetes Patients
Source: Brain Sci. 2022 Dec 12;12(12):1704. doi: 10.3390/brainsci12121704 (PMC9775627; doi:10.3390/brainsci12121704)
Supplement: Supplementary file 1 [file brainsci-12-01704-s001.zip › brainsci-2034447-Supplementary Material.pdf]

Table S1. Association between serum uric acid and DPN in all patients and different subgroup respectively, using restricted cubic splines with 3 knots (at the 10th, 50th, and 90th percentiles).

	P-overall	P-nonlinearity
All patients	0.0504	0.6838
Male subgroup	0.0094	0.4455
Femal subgroup	0.9186	0.6840
Younger subgroup	0.0324	0.4385
Older subgroup	0.5350	0.6212

Table S2. Multivariate linear regression analysis of the relationship between serum uric acid and nerve conduction parameters in the female subgroup and older subgroup.

	Female subgroup **(n** =243**)**		Older subgroup **(n** =198**)**	
	β(95% CI)	p	β(95% CI)	p
MNCV	-0.002[(-0.009)-(0.004)]	0.505	-0.003[(-0.008)-(0.002)]	0.227
F-wave minimum latency (ms)	0.001[(-0.006)-(0.008)]	0.871	0.007(0.001-0.014)	0.028
Ulnar Sensory amplitude (mv)	0.003[(-0.028)-(0.035)]	0.830	0.004[(-0.018)-(0.027)]	0.703
Motor CV (m/s)				
Ulnar	-0.004[(-0.012)-(0.005)]	0.419	0.000[(-0.008)-(0.008)]	0.996
Median	-0.003[(-0.012)-(0.005)]	0.459	-0.002[(-0.009)-(0.004)]	0.443
Tibial	-0.002[(-0.010)-(0.006)]	0.673	-0.007[(-0.013)-(0.000)]	0.036
Common peroneal	0.000[(-0.007)-(0.007)]	0.997	-0.002[(-0.007)-(0.004)]	0.548

The model was adjusted for age, sex, duration of diabetes, glycated hemoglobin, body mass index, smoking, hypertension, total cholesterol, triglyceride, high-density lipoprotein cholesterol, low-density lipoprotein cholesterol, and free thyronine (FT4).

MNCV, mean motor nerve conduction velocity; CV, conduction velocity; CI, confidence interval.

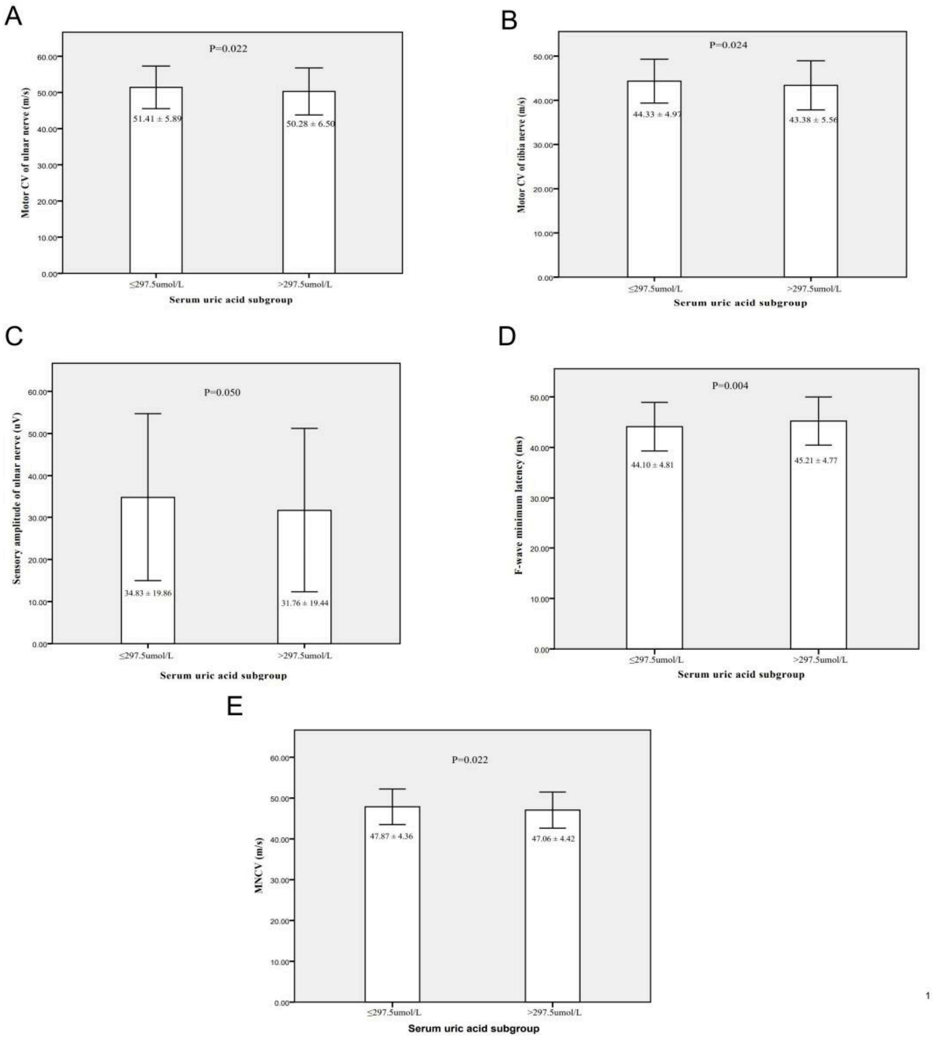

Figure S1. Comparison of the Nerve conduction studies parameters between subgroups based on the optimized cut-off of serum uric acid (SUA) level. Abbreviations: CV, conduction velocity; MNCV, mean motor nerve conduction velocity.
